# Outcomes of a multifaceted intervention to improve maternal satisfaction with care in secondary hospitals in Nigeria

**DOI:** 10.1080/16549716.2020.1856470

**Published:** 2020-12-18

**Authors:** Friday E. Okonofua, Lorretta Favour C. Ntoimo, Bola F. Ekezue, Victor Ohenhen, Kingsley Agholor, Brian Igboin, Kenneth Maduako, Wilson Imongan, Yagana Gidago, Hadiza Galadanci, Rosemary Ogu

**Affiliations:** a Women’s Health and Action Research Centre (WHARC), Benin City, Nigeria; b The Centre of Excellence in Reproductive Health Innovation, University of Benin, Benin City, Nigeria; c The Department of Obstetrics and Gynaecology, University of Benin and University of Benin Teaching Hospital, Nigeria; d Department of Demography and Social Statistics, The Federal University, Oye-Ekiti, Nigeria; e Department of Accounting, Finance, Healthcare Administration & Information Systems, Fayetteville State University, Fayetteville, USA; f Department of Obstetrics and Gynaecology, The Central Hospital, Benin City, Nigeria; g Department of Obstetrics and Gynaecology/Anti-Retroviral Therapy Centre, The Central Hospital, Warri, Delta State, Nigeria; h Department of Obstetrics and Gynaecology, The General Hospital, Minna, Nigeria; i Department of Obstetrics and Gynaecology, The Bayero University, Kano, Nigeria; j Department of Obstetrics and Gynaecology, University of Port Harcourt, Rivers State, Nigeria

**Keywords:** Maternal satisfaction, maternal mortality, referral hospitals, intervention, Nigeria

## Abstract

**Background**: Data in Nigeria suggests a high level of dissatisfaction among women attending maternity care in health facilities due to long wait times, disrespectful care, and poor attention by healthcare personnel.

**Objective**: To examine the effectiveness of a multifaceted intervention in improving self-reported indicators of maternal healthcare satisfaction by women who use referral facilities in two regions of Nigeria.

**Method**: The design was quasi-experimental and consisted of two intervention facilities and two control facilities. The interventions included strategic planning, staff re-training, a computerized appointment system, health education/feedback, maternal death reviews and surveillance, and advocacy. A random sample of 2262 women was selected (1205 in the intervention sites and 1057 in the two control sites) to respond to a 24-item questionnaire on service satisfaction as they exited the health facilities. Adjusted Poisson and binary regression analyses were used to assess and compare proportions of reported satisfaction by women between the intervention and control sites.

**Results**: Women in the intervention sites were 54% more likely than those in control sites to report overall satisfaction with services. They were significantly less likely to report inadequate security arrangements in accessing the health facilities (p < .1); and three times more likely to agree that health workers were extremely thorough and careful in attending to them (p < .1).

**Conclusion**: The interventions had positive effects on improving women’s satisfaction with care. The findings from this study have implications for the design and implementation of interventions that address women’s concerns relating to the provision of care and consequently improve service utilization.

## Background

The reduction of maternal mortality is an important component of the Sustainable Development Goal (SDG) 3 [1]. With high rates of maternal mortality, sub-Saharan Africa remains the region most challenged in achieving this goal [2]. Increasingly, limited access to skilled maternal health care has been recognized as the principal determinant of high maternal mortality rates in the region [2–5]. In Nigeria, with a maternal mortality rate of 814/100,000 births [1], the results of the 2018 Demographic and Health Survey indicate that only 43% of women receive care from a skilled provider during delivery, while 67% attend antenatal care with a skilled provider [6].

Among several reasons proposed to explain the limited use of skilled birth attendants in Nigeria, women’s dissatisfaction with services offered in health facilities was predominant [7–9]. Reports from several developing countries indicate that high rates of maternal dissatisfaction with maternal health services are reasons for non-use of services [10–14]. Some of the concerns expressed by women regarding satisfaction versus dissatisfaction of maternal healthcare include the physical environments and hygiene of health facilities, accessibility of services, interpersonal relationships with healthcare workers, the way health services are organized, and the expertise/competence of healthcare personnel [14–17].

Our previous formative research in Nigeria revealed that a large proportion of women reported being dissatisfied with services offered in health facilities [8,11]. Concerns expressed revolved around the poor attitude of staff, long waiting times, poor attention to women in labor, high cost of services, and sub-standard facilities. Further studies established that women wait inordinately long periods before they obtain services in some referral health facilities [16], due largely to inadequate staffing and heavy workloads by staff [17,18].

While there is ample report of women’s dissatisfaction with maternal health in many African countries [15,19,20], there are limited publications of interventions addressing this challenge [21,22]. In the assessment of the effects of a Community Health Worker (CHW) intervention with a specific focus on maternal and child health in Tanzania, satisfaction with the public health system in Dar es Salaam and satisfaction with the CHW program were key indicators. The respondents (currently pregnant and women who just delivered) in the intervention sites reported significantly higher satisfaction than women in the control sites [21]. Satisfaction with delivery care was one of the outcome indicators in the Saving Mothers, Giving Life intervention program in Uganda and Zambia. The respondents in the intervention arm did not report higher satisfaction with delivery care than women in the comparison sites [22]. A focus group discussion with pregnant women at the formative phase of this study identified specific recommendations that women considered necessary to increase their satisfaction with maternity care [8]. These included the improvement of hospital facilities, reductions in delays in attending to patients, re-training of health workers on providing respectful maternal care, and appropriate counseling of women. In particular, they recommended the systematic reorganization of maternal health services to create an empathic, friendly, and responsive health care system that addresses the needs of women [8].

We addressed these concerns in the intervention we assessed in this study, conducted in referral hospitals over two years. We hypothesized that a multifaceted intervention that addresses all concerns is better suited to improve women’s satisfaction with care rather than a single action intervention. Therefore, we implemented a set of multifaceted (complex) intervention in two referral hospitals in two regions of Nigeria, and two hospitals of comparable status in similar regions served as the control sites. This paper aims to report the effectiveness of the interventions in improving self-reported indicators of service satisfaction by women, one of the outcome indicators of the intervention. We believe the results have implications for designing policies and programs for improving women’s access to skilled maternity care in Nigeria and other parts of sub-Saharan Africa.

## Methods and measures

### Setting

The study is drawn from an intervention research aimed at improving the quality of maternity care in Nigerian referral hospitals. Baseline data were collected from eight referral hospitals, the details of which have been reported elsewhere [8,23]. The intervention was a quasi-experimental study, with the intervention activities implemented over 24 months in two hospitals, while two hospitals were the control sites.

### Intervention vs. control hospitals

The Central Hospital in Benin City, South-South Nigeria, and the General Hospital in Minna, Niger State, in the North-Central region, were the intervention hospitals, while Central Hospital Warri, South-South Nigeria, and the Suleja General Hospital, Abuja, in the North-Central region were the control hospitals. The four hospitals serve large populations of women in two geopolitical zones of Nigeria.

### Intervention activities

In 2014, the Women’s Health and Action Research Centre (WHARC), Benin City, Nigeria, constituted a research team that received a grant from the World Health Organization to conduct implementation research for improving the quality of obstetric care for the prevention of maternal and perinatal mortality in referral hospitals in Nigeria. The formative research phase of the research was completed in December 2015, and the report was documented in March 2016. The results were presented at a workshop for stakeholders such as policymakers, facility managers, and to solicit inputs for the design of the intervention phase of the study. Using the inputs from stakeholders, the final intervention activities were designed by the research team. All the intervention activities started in October 2017 and ended in June 2019, except strategic plan development, which started three months earlier. In all, the intervention period was 24 months.

1) The development and implementation of a strategic plan in collaboration with health providers and policymakers responsible for policy oversight: This activity took place over three months before the commencement of the intervention. The plan included shared strategies for improving women’s satisfaction with maternity care, followed by workshops where we disseminated copies of the strategic plan to staff.

2) Staff re-training: We delivered three-day workshops on knowledge and skills training for doctors and midwives. The training focused on providing quality maternity care, including training on respectful and non-abuse care, proper counseling of women, the use of treatment algorithms for clinical decision-making, prompt attention to patients to reduce delays, and the management of pregnancy complications. Multimodal learning methods used were lectures, discussions, role-plays, demonstrations, and hands-on sessions.

3) Computerized appointment system: We implemented a computerized automated appointment system to reduce the time spent by women during visits to the hospital. Each woman pre-scheduled for a visit received automatic multiple reminders to reduce missed visits and to improve pre-visit planning by the doctors. The appointment system enabled the doctors to see the patients as soon as they arrived in the hospital.

4) Implementation of a composite health education program: A redesigned health education program for pregnant women and their partners was delivered outside clinic hours to reduce the time spent on providing health education to women on clinic days. Monthly health talks were delivered by experts, with health providers and policymakers in attendance. We shared Behavioral Change Communication materials to pregnant women and their partners. The booklet was translated to the Hausa language for those who speak Hausa only.

5) Maternal death reviews and surveillance: Staff of the intervention hospitals was trained to use the Federal Ministry of Health protocol for conducting maternal death reviews and surveillance (MDRS), as described elsewhere [24]. All maternal deaths occurring in the hospitals during the period were reviewed to determine the causes of death. After that, specific remedial measures were taken to correct the deficiencies in clinical management that led to the deaths.

6) Advocacy to policymakers and hospital administrators: We paid regular advocacy visits to policymakers and health administrators responsible for providing services in the two Hospitals. Advocacy focused on resource mobilization and the need to provide an adequate budget for improving service delivery in the hospitals.

## Data

Data were collected for 21 months using a questionnaire.All women who used the intervention and control hospitals for maternal care (antenatal, intrapartum, or postnatal care) during the study period were eligible for inclusion in the study. The exclusion criteria included women who used the intervention and control hospitals for services not related to maternal care, met the inclusion criteria but declined to be interviewed, and do not speak English, Nigerian Pidgin English, or the dominant local language. Using the Yamane formula [25], a sample of 2400 patients was estimated, with 800 participants projected for each hospital. The respondents were approached as they exited the facility, and all consenting women were interviewed. The questionnaires were administered face-face-face by trained staff in English, Pidgin English or the dominant local language using Computer-Assisted Personal Interviewing (CAPI) technique. The CAPI was set up on electronic tablets using the Census and Survey Processing System (CSPro), a public domain software package for entering, editing, tabulating census, and survey data. The data collectors were persons who did not participate in delivering care to women to reduce bias.

In total, 2262 exit interviews were conducted, comprising 777 for antenatal care, 752 for intrapartum care, and 733 for postnatal care. The non-response rate was 5.8%. The exit interview questionnaire was adapted from Health Results-Based Financing Nigeria 2017 Exit Interview questionnaire used by the World Bank, Federal Ministry of Health, and National Bureau of Statistics [26]. The questionnaire assessed individual and family characteristics, parity, antenatal care, childbirth, and postnatal care, and measured the extent to which they were satisfied with care.

### Variables and measures

The outcome variable, satisfaction with care, was measured in two different ways. First, the respondents were asked, ‘overall, would you say you are satisfied with your experience in this hospital.’ Not satisfied and uncertain responses were coded 0, while satisfied and fairly satisfied were coded 1. Second, the respondents were asked questions on 24 items addressing six dimensions: physical access, hospital environment, respectful care (comprising interpersonal and communication skills of the providers and equity), financial cost, waiting time, and technical skill. The importance of assessing care satisfaction with these measures has been documented [12,27]. The response options for the 24 items were ‘disagree,’ ‘neither agree nor disagree,’ and ‘agree.’ Each item was dummy coded: the positive response (indicating satisfaction) was coded 1, whereas the negative and neutral responses were re-coded 0. For further analysis, the 24 items were summed using principal component analysis (scale reliability coefficient was 0.86). The month of the interview, type of treatment (antenatal, intrapartum,or postnatal care), socio-demographic characteristics (age, number of children, marital status, the highest level of education, religion, and occupation), were included as control variables in the final model if significant in bivariate analysis.

## Data analysis

The characteristics of the respondents for each arm were described using summary statistics, frequency, and percentages as applicable. The difference between sites was examined using the non-parametric alternative of t-test (Mann-Whitney test) for continuous variables and Chi-square test for categorical variables. The likelihood of being satisfied overall was estimated using adjusted binary logistic regression, and Poisson regression was used to determine the likelihood of reporting the sum of the 24 items on satisfaction. To compare responses in each of the 24 items by site, a logit model that adjusted for statistically significant characteristics of the respondents and month of the exit interview was estimated with each item as the dependent variable and site as the independent variable. The net effect of each independent variable on overall satisfaction with care was estimated using a logit model for the intervention and control site, adjusting for personal and other statistically significant characteristics of the respondents and the time of the interview. This analysis was necessary to compare the effect of various independent variables on overall satisfaction with care. All the analyses were conducted with Stata13 for windows. The results of logistic regression were presented as odds ratios (OR), whereas the Poisson regression result was presented as incidence rate ratios (IRR) with their 95% confidence interval (CI). All the analyses were two-tailed, and the p-value was set at 0.05.

## Results

### Characteristics of respondents

The characteristics of the respondents are presented in Table 1. A total of 2262 exit interviews were conducted, 53.3% at the intervention sites, and 46.7% at the control sites. As shown in the unadjusted bivariate result, women in the control sites were significantly older than those in the intervention sites. Most respondents had secondary and higher education in both sites. The average number of children per respondent in each site was slightly above two (M = 2.1, SD = 1.6), and the difference between sites was statistically significant. The majority of the respondents were in a marital union in both sites, but the control sites had more respondents who were not in a union. The relationship between marital status and sites was not statistically significant. The distribution of the respondents by religion showed that the majority of the respondents in both sites were affiliated with Christian denominations, but slightly significantly more women in the intervention sites were Muslims. About 48% of the respondents in the intervention sites compared to 60% at the control sites were self-employed, and the proportion of respondents by type of treatment was similar between the sites.

**Table 1.** Characteristics of the respondents

Characteristic	Intervention n (%)	Control n (%)	p-value
Number of respondents	1205(53.3)	1057(46.7)	
Age Range Mean/standard deviation	15–49 29.6 ± 5.9	17–43 30.4 ± 4.8	< .1
Highest level of education No education Primary Secondary Higher	112(9.3) 143(11.9) 457(37.9) 493(40.9)	27(2.5) 98(9.3) 481(45.5) 451(42.7)	< .1
Number of children Mean/standard deviation	2.1 ± 1.6	2.3 ± 1.6	< .1
Marital status Not in union In union	25(2.1) 1180(97.9)	34(3.2) 1023(96.8)	0.089
Religion Catholic Other Christian denominations Islam	133(11.0) 664(55.1) 408(33.9)	201(19.0) 701(66.3) 155(14.7)	< .1
Occupation Not working Civil servant Self-employed Private sector employee	329(27.3) 182(15.1) 582(48.3) 112(9.3)	243(23.0) 98(9.3) 635(60.1) 81(7.7)	< .1
Type of treatment Antenatal care Intrapartum care Postnatal care	427(35.4) 390(32.4) 388(32.2)	350(33.1) 362(34.3) 345(32.6)	0.468

### Maternal satisfaction with care

Relative to respondents at the control sites (Table 2), satisfaction with overall experience of care was significantly higher at the intervention sites (OR 1.54, CI: 1.24–1.91). When all the 24 statements on dimensions of care were summed, the incidence rate ratio was significantly higher at the intervention compared to the control sites (IRR 1.09 CI: 1.07–1.11).

**Table 2.** Comparative analysis of satisfaction with care between and within sites

	Satisfaction with care	Determinants of satisfaction with care		
Variable	Control(Ref) vs. Intervention	Both sites	Intervention site	Control site
**Sum of satisfaction indicators IRR (95% CI)** Range 1–24; mean 19.1 sd 4.5	1.09(1.07–1.11)***			
	**OR (95% CI)**	**OR (95% CI)**	**OR (95% CI)**	**OR (95% CI)**
**Satisfaction with overall experience**	1.54(1.24–1.91)***			
**Physical Access**				
Convenient to travel to facility (Agree)	1.23(0.99–1.52)	1.00(0.74–1.34)	0.93(0.57–1.51)	1.16(0.76–1.76)
Hours the facility is open are adequate to meet your need (Agree)	2.83(2.18–3.69) ***	1.77(1.25–2.51)**	1.82(0.97–3.40)	1.59(0.99–2.56)
Level of security in the hospital area makes it difficult for people (disagree)	0.75(0.62–0.90)**	1.14(0.86–1.52)	0.80(0.52–1.22)	2.44(1.52–3.93)***
**Hospital Environment**				
Facility clean (Agree)	1.22(1.00–1.50)*	1.84(1.36–2.48)***	2.01(1.27–3.20)**	1.55(1.00–2.40)*
**Interpersonal and Communication Skill (Respectful Care)**				
Health workers are courteous and respectful (Agree)	2.09(1.69–2.58)***	1.25(0.91–1.71)	1.46(0.90–2.37)	1.22(0.77–1.92)
Health workers did a good job of explaining patient’s condition (Agree)	2.59(1.95–3.45)***	1.22(0.87–1.72)	1.11(0.57–2.14)	1.35(0.88–2.07)
Had enough privacy (Agree)	3.44(2.69–4.40)***	2.26(1.65–3.10)***	3.33(1.90–5.82)***	2.07(1.367–3.14)**
The health workers are very friendly and approachable (Agree)	3.01(2.42–3.75)***	1.50(1.07–2.10)*	1.75(0.96–3.19)	1.43(0.92–2.24)
The health workers are easy to make contact with (Agree)	4.39(3.57–5.40)***	1.32(0.95–1.81)	1.96(1.11–3.48)*	0.92(0.59–1.42)
The health workers care about your health just as much or more than you do (Agree)	1.87(1.54–2.27)***	1.03(0.78–1.34)	1.65(1.07–2.52)*	0.76(0.52–1.10)
The health workers act differently towards rich people than towards poor people (Disagree)	1.52(1.26–1.83)***	1.51(1.12–2.02)**	1.46(0.95–2.25)	1.911.20–3.02)**
**Financial Cost of Care**				
Registration fee is reasonable (Agree)	0.76(0.54–1.07)	1.09(0.71–1.66)	1.15(0.61–2.14)	0.74(0.37–1.46)
Lab fees reasonable (Agree)	0.26(0.20–0.35)***	1.01(0.69–1.49)	1.06(0.62–1.82)	1.31(0.68–2.54)
Medication fees reasonable (Agree)	0.20(0.15–0.26)***	1.43(0.99–2.08)	0.93(0.56–1.56)	2.56(1.32–5.02)**
Transport fees reasonable (Agree)	0.81(0.62–1.06)	1.05(0.74–1.50)	1.45(0.87–2.43)	0.58(0.33–1.01)
**Waiting time**				
Waiting time to see health provider was reasonable (Agree)	0.90(0.73–1.10)	2.10(1.61–2.75)***	1.33(0.87–2.05)	2.28(1.55–3.34)***
**Technical Skill**				
Easy to get medicine health workers prescribed (Agree)	0.96(0.77–1.21)	1.52(1.13–2.06)**	1.20(0.73–1.96)	1.65(1.09–2.51)*
Health worker spent sufficient time with patient (Agree)	6.47(4.78–8.75)***	0.99(0.68–1.45)	2.01(0.94–4.27)	1.00(0.61–1.64)
The facilities in the hospital are adequate (Agree)	1.26(1.04–1.52)*	1.44(1.09–1.90)*	1.40(0.90–2.18)	1.28(0.86–1.90)
The health workers are extremely thorough and careful (Agree)	3.28(2.54–4.25)***	1.07(0.75–1.52)	1.15(0.61–2.19)	1.09(0.68–1.76)
Trust the skills and abilities of the health workers (Agree)	7.57(5.13–11.18)***	0.72(0.49–1.07)	1.11(0.37–3.35)	0.77(0.50–1.19)
Completely trust the health worker’s decisions about medical treatment (Agree)	6.11(4.15–8.98)***	0.84(0.58–1.22)	0.51(0.18–1.45)	0.99(0.64–1.52)
Overall quality of services satisfactory (Agree)	2.31(1.76–3.04)***	2.14(1.52–3.02)***	3.57(1.90–6.71)***	1.60(1.03–2.49)*
All in all, you trust the health workers completely (Agree)	2.46(1.70–3.54)***	2.17(1.42–3.33)***	2.52(1.05–6.07)*	1.73(1.03–2.90*

Note: ***p < .1, **p < .1, *p < .5

The odds of satisfaction in two of the three physical access factors were significant at the intervention compared to the control sites. The likelihood of agreeing that ‘the opening hours is adequate to meet their needs’ was significantly higher at the intervention site (OR 2.83 CI:2.18–3.69), and disagreeing that the level of security in the hospital area makes it difficult for people to access the hospital was 25% lower at the intervention sites. Satisfaction with the hospital environment, which was measured with whether the hospital was clean, was significantly higher at the intervention site (p < .5)

Respectful care was measured with seven items of providers’ interpersonal and communication skills. In all of the seven items, the odds of satisfaction were higher at the intervention compared to the control sites. For instance, the odds of agreeing that the health workers are courteous and respectful were 2.09 times higher at the intervention site. The odds of reporting that laboratory fees and medication fees were reasonable were 74% and 80% less likely, respectively, at the intervention site.

Technical skill, which included clinical competency and overall assessment of the quality of care, was measured with eight items. The likelihood of satisfaction was significantly higher at the intervention site in seven of the items. For instance, the odds of agreeing with the statement that the health workers were extremely thorough and careful was 3.28 times higher at the intervention compared to the control sites.

### Determinants of maternal satisfaction with care

Analysis of the net effect of potential determinants of overall satisfaction for both sites and within intervention and control sites separately, is presented in Table 2. The combined data for both sites show that out of the three physical access factors, only adequacy of opening hours was a significant determinant of satisfaction with care (OR 1.77 CI:1.25–2.51). The physical environment factor measured by cleanliness of the facility was also a significant determinant. Of the respectful care factors, enough privacy, providers being friendly and approachable, and health providers not acting differently towards rich and poor people were significant predictors of overall satisfaction with care. Waiting time considered as reasonable was a further statistically significant determinant. Out of all measures of technical skills, getting prescribed medicine easily, adequacy of hospital facilities, satisfactory quality of services, and complete trust in the health workers were significant predictors of overall satisfaction.

Results of the within site analysis show that none of the physical access items was significant at the intervention site, but security in the hospital area was a significant factor at the control site. The hospital environment cleanliness was an important determinant in both sites. Respectful care in the form of privacy was significant in both sites; easy contact with health providers, health workers care about respondent’s health were significant determinants at the intervention site, whereas the issue of providers equally treating all patients, was a significant factor at the control site. The financial cost was not a significant predictor at the intervention site, but medication fees were significant for respondents at the control site. Waiting time was not a determinant at the intervention site, but it was at the control site where respondents who agreed that waiting time was reasonable were more likely to be satisfied overall (OR 2.28 CI: 1.55–3.34). Concerning technical skills, getting the medicines prescribed by health workers easily was a significant determinant at the control site, but overall satisfaction with the quality of services and complete trust in health workers were significant at both intervention and control sites.

## Discussion

The objective of this study was to determine whether a set of multifaceted intervention activities would be effective in improving the satisfaction of women with maternal healthcare services in referral facilities in Nigeria. The results show that women in intervention sites were 54% more likely to report satisfaction with services compared to women in the control sites. In this study, we measured the level of satisfaction with a 24-item questionnaire that was sub-divided into three categories. These included questions relating to the physical environment, access to the hospitals, and respectful maternity care/staff-client interactions. The results showed that women in the intervention facilities were 22% more satisfied with the physical environment than women in the control sites. For physical access, the results show that women in the intervention sites were nearly three times more likely to report that the opening hours at the facilities meet their needs. They were also significantly less likely to report that the security arrangements in the health facilities posed a challenge to them in accessing the facilities. The finding for assessment of respectful care also showed better results in the intervention sites compared to the control sites. Women in the intervention sites were twice more likely to report that health workers are courteous and respectful. Similarly, women in intervention sites were at least three times more likely to agree that health workers were extremely thorough and careful compared to women in the control sites.

These favorable results may be attributable to the composite nature of our interventions and the fact that intervention components were identified after intense formative research in collaboration with women and other relevant stakeholders. Multifaceted interventions that simultaneously address various barriers has been shown to improve some health outcomes in sub-Saharan Africa [28–30]. However, the Saving Mothers, Giving Life initiative in Uganda and Zambia, which involved multiple intervention activities that addressed demand, access, quality, and systems strengthening, did not result in a high report of satisfaction by the women [22].

Our formative research included interviews with health managers in the referral hospitals [31] that were experiencing high rates of maternal mortality due to deficits in the health system. After that, we worked with hospital managers and staff to develop and implement a strategic plan that envisioned the reduction in maternal mortality ratios within one year. We believe that the strategic plan re-positioned the intervention hospitals to be more cohesive and efficient in improving the quality of maternal health care. Existing laws, policies, and practices relating to the prevention of maternal mortality, such as MDRS, were actively carried out during the period.

This study indicates that the improvement of maternity care and prevention of maternal mortality requires a collaborative approach and the participation of committed stakeholders. To the best of our knowledge, this is one of the first reports of the development and implementation of an institutional strategic plan in Nigeria aimed at reducing maternal mortality in health institutions. We believe this approach is scalable and can improve the quality of all elements of maternal health care in referral hospitals in Nigeria, and can be adopted by other low-resource countries.

Further analysis of the data showed that the most important determinants of women self-reporting of satisfaction with care were: adequacy of opening hours at the facilities, cleanliness of the physical environments, providers being friendly and approachable, adequacy of facilities, medicines being readily available, and not waiting too long to receive care. Most interestingly, social equity turned out to be an essential expectation by women, an expectation that rich and poor women were treated equally without differential attention because of financial status. This was an important issue that the women in both intervention and control sites considered critical to their satisfaction with health care. Equitable and respectful care as a predictor of satisfaction with health care has been shown in previous studies [14,19]. We recommend that health providers and policymakers consider these preferences by women in their design of women-friendly maternal health care services to increase maternal satisfaction and utilization.

Of interest was the finding that financial consideration was not a significant predictor of women reporting satisfaction with care in the intervention sites. This suggests that when other factors are satisfactory, women will not consider costs as burdensome. This finding is supported by previous reports that indicate that cost consideration is secondary to other indicators of quality of care [11,14]. However, some reports suggest cost an essential factor to women in their preference for services [32,33].

To date, there has been limited evidence of effective interventions that promote women’s satisfaction with maternal health care in African countries. The World Health Organization reported a single cluster randomized trial that concluded that interventions might have little or no effect on women’s satisfaction with maternal health care [34]. Further studies did not evaluate interventions aimed at changing practice culture, such as those presented in this current study [7,35–37]. Therefore, this study provides one of the first empirical evidence indicating that effective interventions can promote women’s satisfaction with maternal health care in Nigeria.

### Strength and limitations of the study

The major strength of the study is the comprehensive and multifaceted nature of the intervention, which addressed key concerns on satisfactory care raised by women in prior research. Also, involving multiple stakeholders in the project development and implementation and the large sample of women interviewed during the exit interviews enabled the collection of varied perspectives. However, the study was confined to four hospitals in only two out of the country’s six geopolitical zones, which is a limitation. Our desire to ensure effective implementation of the project activities and accurate data collection, prompted us to limit the scope of the project. Nevertheless, we believe that the results are generalizable to the rest of the country, as the participating health facilities serve large populations of women in two major regions of the country.

## Conclusion

We conclude that interventions that addressed women’s multiple concerns on the provision of care and implemented in a multi-disciplinary manner with the involvement of facility managers and policymakers are effective in improving women’s satisfaction with maternal health care. The results have implications for the design of policies and programs to improve the quality of maternal health care services in all parts of Nigeria and other less developed countries.

## Paper context

Poor satisfaction with maternal health services has been indicated as a strong reason for the non-use of services in Nigeria. We conducted formative research in eight referral facilities to determine women’s perceptions and experiences with care. We then used data from the formative research to design the components of the intervention. Our findings suggest a higher value women attribute to various quality and satisfaction indicators other than costs, which can inform policies.
